# Acupuncture for arousal combined with repetitive transcranial magnetic stimulation promotes functional reorganization of brain regions in patients with a minimally conscious state: study protocol for a randomized controlled trial

**DOI:** 10.3389/fneur.2025.1610462

**Published:** 2025-08-04

**Authors:** Jiajia Li, Hongjing Fang, Tong Liu, Peixin Li, Zhihong Ou, Zhihui Li, Tao Huang, Juan Sun, Jueyu Zhang, Fangyi Lu, Xi Wen

**Affiliations:** ^1^Department of Acupuncture and Rehabilitation, The Fifth College of Clinical Medicine, Guangzhou University of Traditional Chinese Medicine, Guangzhou, China; ^2^Department of Acupuncture and Rehabilitation, Guangdong Second Hospital of Traditional Chinese Medicine, Guangzhou, China; ^3^Department of Emergency, North Guangdong Second People’s Hospital, Shaoguan, China

**Keywords:** acupuncture, transcranial magnetic stimulation, disorders of consciousness, randomized controlled trial, protocol

## Abstract

**Introduction:**

The minimally conscious state (MCS) is one of the common and serious complications of stroke patients. Its incidence is higher than that of other types of consciousness disorders. The prolonged monitoring and comprehensive management of patients with MCS imposes substantial socioeconomic burden, creating multifaceted challenges for healthcare systems and considerable strain on familial resources. However, there are still few studies on the treatment of MCS, so there is a lack of standardized and effective treatment for MCS, which needs to be solved urgently. In order to solve this problem, this study proposes a treatment plan of acupuncture for arousal combined with repetitive transcranial magnetic stimulation (rTMS). Acupuncture for arousal is a clinical acupuncture therapy. Accumulating evidence from the published literature indicates that acupuncture for arousal interventions can significantly enhance consciousness levels in comatose patients. Nevertheless, conventional acupuncture monotherapy necessitates extended treatment durations, potentially compromising patient outcomes and neurological recovery trajectories. rTMS can excite or inhibit specific cortical functional areas of the brain, thereby improving the level of consciousness in comatose patients. The significance of this study is to clarify the efficacy and safety of wake-promoting acupuncture combined with repetitive transcranial magnetic stimulation in patients with minimal consciousness after stroke, and to add a standardized and effective scheme for the treatment of MCS. Furthermore, this investigation employs neural-specific biomarkers and functional magnetic resonance imaging to elucidate the mechanisms underlying regional brain functional reorganization. These neuroimaging and molecular approaches may establish a robust scientific foundation for future investigations into consciousness-restoring mechanisms in patients with MCS.

**Methods and analysis:**

This is a single-center design, randomized, controlled, third-party blind trial involving 117 patients. Three groups of patients will receive 4 weeks of treatment. Under basic treatment, the acupuncture for arousal group will only receive acupuncture treatment. The rTMS group only receives rTMS treatment; the combined group is treated with the acupuncture for arousal and rTMS. The main outcome indicators are the GCS score scale, the CRS-R score scale, central nerve-specific protein (S-100B), neuron-specific enolase (NSE), and functional magnetic resonance imaging (fMRI). The secondary outcome indicators are laboratory safety parameters, including routine blood tests, routine urine tests, coagulation tests, electrocardiograms, liver function tests, and renal function tests. Finally, the measurement data and enumeration data will be analyzed via SPSS 25.0 software.

**Ethics and dissemination:**

This study has been approved by the Ethics Committee of the Second Chinese Medicine Hospital of Guangdong Province (Approval No.: K202405-003-01, June 19, 2024). The research results will be published in peer-reviewed journals.

**Clinical trial registration:**

https://www.chictr.org.cn/bin/project/edit?pid=235202, ChiCTR2400086163.

## Highlights

- This study is the first to combine a traditional therapy (acupuncture for arousal) with a modern neuromodulation technique (repetitive transcranial magnetic stimulation, rTMS), providing a novel multimodal treatment regimen for the patient population (MCS) that currently requires more standardized treatments.
- In terms of efficacy evaluation, the advantage of this study is to adopt a multi-modal assessment approach, through objective data such as clinical behavioral assessment scales, objective biomarkers (NSE, S100B), and BOLD-fMRI, to comprehensively evaluate the patient 's consciousness recovery level, neurological function recovery and brain function reorganization.
- Compared with the previous clinical studies of acupuncture, this study innovatively proposed the acupuncture position of sitting position, because acupuncture at neck acupoints can improve the blood supply of vertebral artery and carotid artery, improve the signal of brainstem reticular ascending activation system, and enhance the compensatory mechanism of the brain. In addition, sitting position can induce patients' proprioception.

## Introduction

The World Health Organization defines stroke as “rapidly developed clinical signs of focal (or global) disturbance of cerebral function, lasting more than 24 h or leading to death, with no apparent cause other than of vascular origin” (1). Disorders of consciousness, including coma and other states of impaired consciousness, represent significant, persistent, and clinically challenging complications frequently observed in the post-stroke population. Related studies have shown that approximately 1/3 of stroke patients can experience varying degrees of disturbance of consciousness (2). Modern medical wake-up therapy includes drug therapy, noninvasive brain stimulation [such as repetitive transcranial magnetic stimulation (3), transcranial direct current stimulation (4), percutaneous auricular vagus nerve stimulation (5, 6), etc.], invasive brain stimulation [deep brain stimulation (7) or vagus nerve stimulation (8)], hyperbaric oxygen therapy and traditional Chinese medicine acupuncture therapy. MCS is characterized by severe consciousness impairment following stroke, wherein patients exhibit profound deficits in awareness and cognitive function while demonstrating reproducible, albeit limited, behavioral evidence of self-awareness or environmental perception (9). The incidence of MCS is 10 times higher than that of persistent vegetative state (10). However, there are significantly fewer studies focusing on MCS than on chronic vegetative state and Coma. Despite extensive investigation into diverse neuromodulatory approaches, including various brain stimulation methodologies, targeted anatomical locations, and rehabilitative behavioral interventions, a standardized, evidence-based therapeutic protocol for MCS remains notably absent from clinical practice. Moreover, there is a lack of in-depth discussion on the mechanism of acupuncture improving MCS consciousness. Therefore, the field of awakening in MCS patients is worthy of further exploration in the future (11).

Patients with cerebrovascular accidents who undergo early initiation of comprehensive rehabilitation protocols demonstrate significantly improved survival rates, enhanced functional independence, and more favorable consciousness recovery trajectories compared to those receiving delayed interventions (12). Acupuncture, as part of its comprehensive rehabilitation strategy, is widely accepted in post-stroke disorders of consciousness in China. Acupuncture for arousal (also known as “cuxing acupuncture” in China) is a clinical acupuncture therapy. It is widely used in clinical practice to treat consciousness disorders caused by various cerebrovascular diseases by using specific acupuncture points and manipulations. Previous studies have shown that acupuncture for arousal can improve the level of consciousness in coma and can upregulate the expression of neurotransmitters related to awakening in coma rats after traumatic brain injury (13). The main reason is that the key acupoints of the acupuncture for arousal are located in the neck. Its arousal-promoting mechanism is considered to be related to improvements in the excitability of the brainstem ascending reticular activating system (ARAS) (14, 15). Notably, in addition to the inhibition of ARAS, MCS patients also exhibit a state of cerebral cortex inhibition to a certain extent. Although the early use of the acupuncture for arousal can improve the level of consciousness of MCS patients with stroke, it takes a long course of treatment, and the ratio of patients who can fully wake up successfully is not ideal. Xia et al. (16) reported that 16 patients with disturbance of consciousness (including MCS and unresponsive wakefulness syndrome) improved their consciousness level after receiving high-frequency rTMS in the dorsolateral prefrontal lobe, especially in MCS patients. It has been reported that rTMS-mediated cortical stimulation may activate, inhibit, or interfere with the activity of neural networks (17). For example, high-frequency rTMS increases the excitability of the motor cortex (18). These suggest that high-frequency rTMS is helpful to improve the patient’s state of consciousness.

NSE has neurotrophic and protective properties for neurons. When neurons are damaged or necrotic, cell membrane integrity is destroyed, blood–brain barrier permeability is increased, and NSE levels in serum and cerebrospinal fluid are increased (19). The level of serum NSE in patients with brain injury is significantly higher than that in normal people. The level of serum NSE can reflect the degree of neuronal damage and play an important role in the early diagnosis, evaluation and prognosis of brain diseases (20). S-100B is a specific protein located in the glial cells of the central nervous system. Its level changes can evaluate the degree of nerve cell injury and death, and it is widely used in the prognosis evaluation of patients with stroke and brain injury (21). S-100B can regulate the proliferation, differentiation and energy metabolism of nerve cells, and the damage and apoptosis of nerve cells can promote the release of S-100B. The increase of S-100B level can activate the related nerve signal transduction, regulate the activity of nervous system, and then predict and diagnose brain injury (22).

Compared with other states of disturbance of consciousness after stroke, the incidence of MCS after stroke is high, and patients often need long-term monitoring and treatment to survive, causing great burden and huge economic pressure to society and families (23). In addition, there is no effective and standardized treatment for MCS, so it is of great economic and social significance to find a safe and effective wake-up method for MCS patients. Lam Ching et al. (24) reported that compared with Western medicine, acupuncture alone or plus rTMS, traditional Chinese medicine, traditional Chinese medicine combined with Western medicine were more effective in improving depression symptoms of patients with post-stroke depression. Among all therapeutic strategies evaluated, the combination of acupuncture with rTMS emerged as the most efficacious intervention modality, suggesting potential synergistic neuromodulatory mechanisms warranting further investigation in controlled clinical trials. Therefore, for patients with post-stroke disturbance of consciousness, we put forward the scientific hypothesis that acupuncture combined with rTMS can promote the improvement of consciousness in patients with MCS. This study will utilize acupuncture for arousal combined with rTMS to treat post-stroke MCS, conducting efficacy and safety evaluations to facilitate its future clinical application and to establish a standardized, effective treatment scheme for MCS. Meanwhile, through the detection of neural specific markers and brain functional magnetic resonance imaging, the mechanism of promoting the functional reorganization of brain regions will be discussed, which could provide a more scientific basis for the study of the wake-promoting mechanism of MCS in the future.

## Methods

### Study design and recruitment

**Figure 1:**
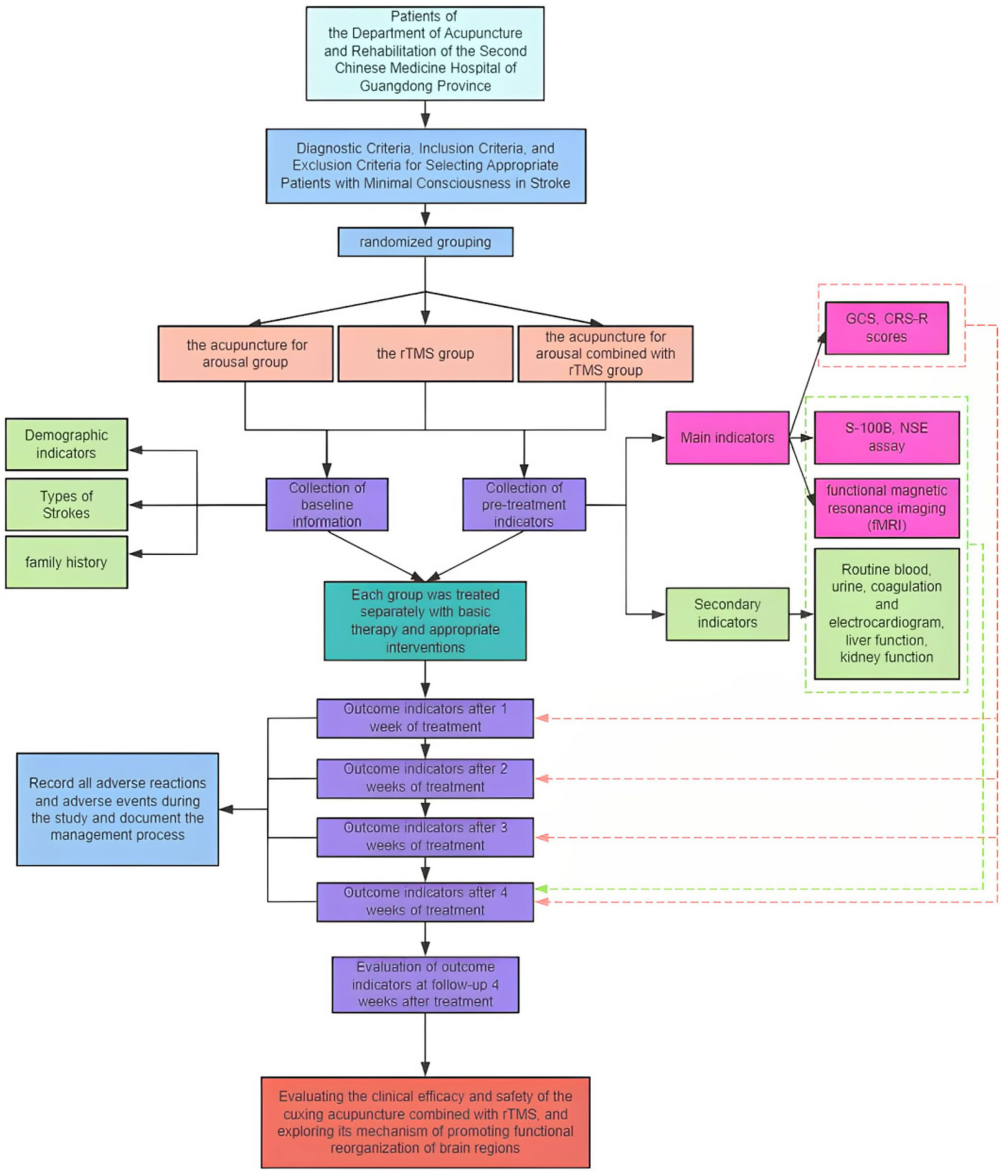
The flowchart of this study. rTMS, repetitive transcranial magnetic stimulation; GCS, Glasgow Coma Scale; CRS-R scores, Coma Recovery Scale-Revised; S-100B, central nervous system-specific protein (S-100B); NSE, neuron-specific enolase.

**Figure 2:**
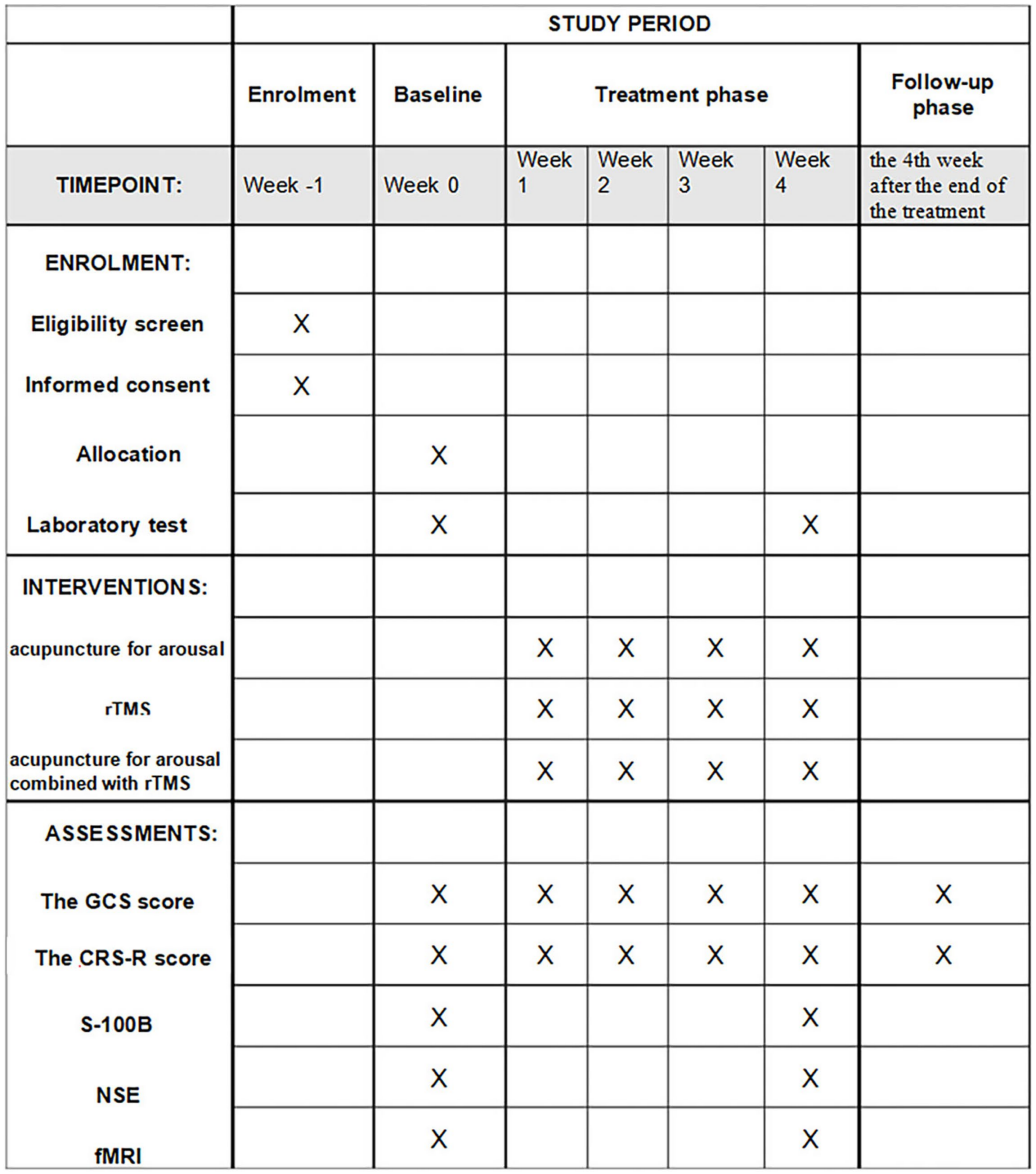
The time points of assessment. rTMS, repetitive transcranial magnetic stimulation; GCS, Glasgow Coma Scale; CRS-R scores, Coma Recovery Scale-Revised; S-100B, central nervous system-specific protein (S-100B); NSE, neuron-specific enolase; fMRI, Functional magnetic resonance imaging.

This is a single-center design, third-party, blinded, randomized controlled trial. Patients with MCS after stroke will be recruited by JJL, HJF, PXL, ZHO and ZHL through the inpatient of the Department of Acupuncture and Rehabilitation of the Guangdong Second Hospital of Traditional Chinese Medicine. A total of 117 patients will be recruited from November 1, 2024, to September 1, 2025. JYZ will use SPSS 25.0 statistical software to generate random numbers. TH and JS will divide the patients into 3 groups, with 39 patients in each group. All of them will be given basic treatment for poststroke wakefulness promotion and the corresponding experimental interventions. The flowchart of this study is shown in Figure 1, and the time points of assessment are shown in Figure 2.

### Patient selection of the study population

When stroke patients are admitted to the Department of Acupuncture and Rehabilitation of the Guangdong Second Hospital of Traditional Chinese Medicine, JJL, HJF, PXL, ZHO and ZHL will use the CRS-R score scale to assess the level of impaired consciousness of the patients. And stroke patients who meet the minimally conscious state (including MCS+, MCS−) in the CRS-R score scale will be screened. TH and JS will screen again and check the information of the study participants to ensure that they meet the inclusion criteria. The criteria of successful recruitment: meet all the inclusion criteria, and do not meet any of the exclusion criteria. The inclusion and exclusion criteria are detailed in Table 1.

**Table 1:** The inclusion and exclusion criteria of the study.

Inclusion criteria	Exclusion criteria
Conform to the diagnostic criteria of stroke and the minimally conscious state. [The diagnostic criteria of cerebral infarction and cerebral hemorrhage are referred to Diagnostic Highlights of Various Major Cerebrovascular Diseases in China 2019 (56). The diagnostic criteria of the minimally conscious state are referred to as The 2018 edition of the American Practice Guide for Disorders of Consciousness (57).]	Those who have participated in other research projects in the last 3 months.
Aged 18–70 years, regardless of gender.	Patients with unstable vital signs (blood pressure, respiration, pulse) or with active cerebral hemorrhage.
Consciousness disorder caused by non-other reasons.	Complicated with serious heart, lung, liver, and kidney complications.
Written informed consent.	Those who do not receive acupuncture treatments.
Duration of impaired consciousness less than 6 months.	Those who are not suitable for magnetic resonance examination because of metal foreign objects in the body, such as coronary stent implantation and pacemaker implantation (58).
	Women in lactation.

Diagnostic Highlights of Various Major Cerebrovascular Diseases in China 2019: Stroke includes cerebral infarction and cerebral hemorrhage, which can be diagnosed by head CT or MRI. The 2018 edition of the American Practice Guide for Disorders of Consciousness: MCS is mainly evaluated by CRS-R. When the patient has a simple command, a “yes or no” response through gesture/language, a clear and understandable language/body expression, and a corresponding action and emotion according to the environment, it can be judged as MCS.

### Written informed consent

JJL, HJF, PXL, ZHO, and ZHL will obtain the informed consent of the patient’s authorized family members. With written informed consent, all participants will be informed that they will be randomly assigned to any group, with possible benefits and risks. And they will be allowed to withdraw at any time during the study.

### Randomization and blinding

SPSS 25.0 software will be used to generate 117 random numbers between 0 and 1 and rank them. They will be marked and divided into 3 groups (the acupuncture for arousal group, rTMS group, and the acupuncture for arousal combined with rTMS group) and made into cards. These random cards will be placed in opaque, sealed envelopes. The patients will extract the cards according to the order of enrollment and be assigned to the corresponding groups according to the contents of the cards.

Owing to the particularity of acupuncture treatment, this study will not implement a double-blind approach and will only implement a blind evaluation of the efficacy. To reduce detection bias, this study’s efficacy evaluation will be completed by professional evaluators who are not involved in the treatment process and do not know the grouping. These evaluators are senior personnel in the field of coma rehabilitation, with standardized behavioral scale assessment certification qualifications. And before the start of the study, all evaluators will be trained and assessed in a unified manner to ensure the homogeneity of the evaluation criteria.

### Interventions

#### Both groups

Baseline data (including stroke type, family history, and demographic information) will be collected from eligible patients. Before treatment, the GCS score and the CRS-R score will be evaluated, and S-100β, NSE, and fMRI data will be measured. The laboratory test items will be detected for safety evaluation (Table 2).

**Table 2:** Comparison of baseline characteristics among three groups (before treatment).

Category		Acupuncture for arousal group (*n* = 39)	rTMS group (*n* = 39)	Acupuncture for arousal combined with rTMS group (*n* = 39)	*p*-value
Demographics		-	-	-	-
Age (years, Mean ± SD)		XX ± XX	XX ± XX	XX ± XX	-
Gender (male/female, *n*)		XX (XX%)	XX (XX%)	XX (XX%)	-
Disease duration (days, Mean ± SD)		XX ± XX	XX ± XX	XX ± XX	-
Comorbidities (HTN/DM, etc. *n*)		XX (XX%)	XX (XX%)	XX (XX%)	-
Stroke type		-	-	-	-
Ischemic stroke (*n*)		XX (XX%)	XX (XX%)	XX (XX%)	-
Hemorrhagic stroke (*n*)		XX (XX%)	XX (XX%)	XX (XX%)	-
Severity		-	-	-	-
GCS score (3–15, Mean ± SD)		XX ± XX	XX ± XX	XX ± XX	-
CRS-R Scale	CRS-R total score (0–23, Mean ± SD)	XX ± XX	XX ± XX	XX ± XX	-
	MCS− subgroup (*n*)	XX (XX%)	XX (XX%)	XX (XX%)	-
	MCS+ subgroup (*n*)	XX (XX%)	XX (XX%)	XX (XX%)	-

Age, Disease duration, GCS score, and CRS-R total score are measurement data, and the data will be presented as “Mean ± SD.” Gender, Stroke type, and MCS subgroup are enumeration data, and the data will be presented as “*n* (%).” *p*-value: The *p*-value come from One-way analysis of variance (measurement data) or Mann–Whitney test (enumeration data). If there is no difference between the three groups, then *p* > 0.05. If there is a difference, the specific *p* is marked. HTN, Hypertension; DM, Diabetes Mellitus.

The three groups of patients will undergo basic treatment and corresponding intervention measures for 4 weeks. First, they will be given basic treatment for awakening after stroke, which mainly includes the following:

1. Drug treatment: For basic diseases (such as hypertension, diabetes and hyperlipidemia, etc.), symptomatic treatment measures are taken, including blood pressure and blood glucose regulation, lipid-lowering and plaque stabilization treatment, anti-platelet aggregation, etc. Concomitantly, the administration of consciousness-altering pharmacological agents with potential neurological modulatory effects should be stringently avoided, encompassing—but not limited to—central nervous system stimulants, cholinergic agonists, and phytotherapeutic/natural compounds possessing arousal-enhancing properties. Furthermore, cerebral metabolic modulators should be systematically excluded from the therapeutic regimen, specifically those that facilitate cerebral bioenergetic processes, enhance neuronal oxygenation or substrate utilization, potentiate neurotransmitter biosynthesis, or exert neuroprotective mechanisms at the cellular or molecular level. There are also prevention and treatment of complications, providing anti-infective drugs to prevent infection, anti-coagulant drugs to prevent thrombosis, drugs to protect the gastric mucosa, and drugs to reduce gastrointestinal complications, etc. And nutritional support treatment, such as providing energy, protein, electrolytes, and vitamin supplements.
2. Specialized nursing: including the placement of functional positions, the correct position can help prevent pressure sores and other problems that may be caused by long-term bed rest. Bladder management and regular emptying of the bladder can prevent urinary tract infections and urinary retention. Artificial airway management, if patients need respiratory support, may need tracheal intubation or other artificial airways. Skin management involves regularly turning over and cleaning the skin and using appropriate mattresses and care products to prevent pressure sores. These will be implemented by a unified professional nursing team.

#### The acupuncture for arousal group

Based on basic treatment, the patients in this group will be treated with acupuncture alone. The positioning of acupuncture points refers to the national standard of the People’s Republic of China in 2021 (GB/T 12346-2021) “Nomenclature and location of meridian points” (GB/T 12346-2021 Nomenclature and location of meridian points [S]). The selected acupoints are bilateral GB20 (Fengchi), GB12 (Wangu), BL10 (Tianzhu), GV15 (Yamen), and GV16 (Fengfu).

Due to the inability of coma patients to sit autonomously, the family members or caregivers will cooperate to fix the patient’s head to take the sitting position. Local acupoints will be disinfected routinely, and Huanqiu brand 0.30 mm × 40 mm and 0.30 mm × 50 mm stainless steel millimeter needles will be used (Suzhou Acupuncture and Moxibustion Supplies Co. Ltd.). Among them, GB12, BL10, GV15, and GV16 use the twirling-reducing method to directly puncture 1 inch; GB20 points to the opposite side of GB20 points through the puncture 1.5 inches. Because the patient is comatose, Deqi is based on the sense of tightness in the hands of the doctor. Then, the BT701-l B-type electroacupuncture instrument (Shanghai Huayi Medical Instrument Co., Ltd.) will be connected, the sparse-dense wave will be selected, the current intensity is 0.4 mA (to the extent that the local impending muscle twitched with the frequency of the pulse but the naked eye did not see the twitching), and the wave will be energized for 20 min, 1 time/day, 6 times/week, and 4 weeks of treatment.

#### The rTMS group

According to the principle of Faraday’s electromagnetic induction, rTMS generates a magnetic field on the coil through a strong current, and then, the magnetic field penetrates the skull into the cerebral cortex without attenuation and causes a local microinduced current in the corresponding cortex, changes the membrane potential of the cerebral cortex, affects brain metabolism and neuroelectric activity, and thus causes physiological and biochemical reactions. Based on basic treatment, the patients in this group will be treated with rTMS alone. The specific operations are as follows.

First, all patients in the rTMS group will have their resting motor threshold (RMT) measured by a physical therapist who is not involved in the current study before the intervention. When the patient’s muscle is in a relaxed state, TMS will be used to stimulate the motor cortex. At least 5 of the 10 stimulations induce the minimum stimulation intensity required for the motor-evoked potential of the target muscle with an amplitude of more than 50 μv, which is the motor threshold we need. The patient will subsequently lie flat on the bed, and the light will be dimmed. The transcranial magnetic stimulation device (Magstim, United Kingdom, Rapid2) will be used, and the midpoint of the intersection of the two coils of the “8”-shaped coil will be placed in the dorsolateral prefrontal cortex corresponding to the scalp according to the international EEG standard, paying attention to the tangency with the scalp. The dorsolateral prefrontal lobe localization method: Use the International 10–20 system-based EEG electrode localization cap, and the “F3” mark on the localization cap is the position of the dorsolateral prefrontal lobe. (Anatomical localization: Taking the left side as an example, the parietal center (the midpoint of the line between the nasal root and the occipital bone) is the starting point, and a vertical line is made at 8 cm from the line with the nasal root, and the left dorsolateral prefrontal lobe is 6 cm to the left.) The parameters used will be as follows: output intensity of 80% of the motor threshold, frequency of 10 Hz, stimulation time of 2 s, string interval of 20 s, 20 min/time, 1 time/day, 6 times/week, and 4 weeks of treatment. All rTMS treatments will be performed by professionally qualified physical therapists. There is no sham rTMS in this study.

#### The acupuncture for arousal combined with rTMS group

Patients in this group will be treated with acupuncture for arousal and rTMS. First, the acupuncture for arousal treatment will be given. After treatment, rTMS treatment will be continued. The operation will be the same as above, with 1 time/day, 6 times/week, and 4 weeks of treatment.

### Criteria for termination of the study

The study will be terminated when the following situations occur: poor compliance of patients after enrollment and non-cooperation with experimental research; during the treatment, critically ill patients need to be transferred to other departments or hospitals for treatment; the patient’s immediate family members request to withdraw from the study.

### Outcome measures

#### Primary outcomes

Our main purpose is to observe the improvement in the level of consciousness in patients with a minimal conscious state after stroke, so we choose some common clinical behavioral assessment scales and brain function test items.

##### The GCS score

The most widely used consciousness assessment tool for patients with disturbances of consciousness (25, 26). The highest possible score is 15 points, indicating clear consciousness; a score of 9 points or above is defined as awakening, and a score of less than 8 points is defined as coma. The lower the score is, the more severe the disturbance of consciousness.

##### The CRS-R score

Patients’ recovery of consciousness is rated on six dimensions, namely, auditory, visual, motor, speech, communication, and arousal, with scores ranging from 0 to 23. The higher the score of CRS-R, the better the patient’s state of consciousness. According to research, the design of the CRS-R is especially suitable for identifying patients with vegetative states and a minimal state of consciousness (27–29). It includes evaluation indicators that the GCS does not have, such as visual tracking and visual positioning. The CRS-R scale can further divide the state of consciousness into MCS -, MCS+, and eMCS. MCS− includes object localization, visual pursuit or fixation, automatic motor behaviors, object manipulation, and localizing noxious stimuli. MCS+ includes the ability to follow commands, object recognition, comprehensible verbal expression and meaningful communication (30). eMCS includes the functional use of two different objects and functional interactive communication (31). Regarding outcome indicator, data collection should include not only longitudinal changes in the CRS-R score but also the specific temporal dynamics of clinically significant consciousness improvement. This is defined as the time for patients with MCS− to eventually improve to MCS+ or eMCS/patients with MCS+ to eventually improve to eMCS after treatment.

##### Detection of central nerve-specific protein and neuron-specific enolase

S-100B is an important index used to evaluate damage to brain nerves, and neuron-specific enolase (NSE) is a type of nerve damage-specific marker that is commonly used in the clinic. The levels of S-100B and NSE can indicate the degree of nerve damage.

##### Functional magnetic resonance imaging

Functional magnetic resonance imaging (fMRI) can reflect the changes in blood oxygenation (BOLD), blood flow changes, and other conditions in the functional areas of the brain and then evaluate brain function stereoscopically and intuitively. BOLD-fMRI imaging can reflect dynamic changes in the functional activity of the cerebral cortex through hemodynamic changes following neuronal activity. A 3.0 T superconducting magnetic resonance imaging system (Siemens) will be used to collect imaging data. The gradient echo plane imaging sequence will be used to collect BOLD-fMRI data. Resting-state functional MRI is acquired at the beginning of the scan, and the parameters are as follows: TR = 2,000 ms, TE = 30 ms, flip angle = 90°, FOV = 224 × 224 mm, matrix size = 64 × 64 mm, slice thickness = 3.5 mm, and slice number = 33 slices. BOLD-fMRI will be performed 30 min before the intervention (baseline period) and 30 min after the end of the 4-week intervention (end period).

#### Secondary outcomes

The secondary outcome measures of this study are the safety test items that can assess the patient’s health status and potential safety hazards. It mainly includes routine blood tests, routine urine tests, coagulation tests, electrocardiograms, liver function tests, and renal function tests. These test items can help researchers understand the health status of patients, detect and address possible health problems in time, and ensure the safety of research.

### Efficacy evaluation

#### The threshold of clinical improvement (Using the CRS-R scale as the reference standard)

Using the type of disturbance of consciousness in the CRS-R scale as the reference threshold for clinical improvement. The clinical significantly effective: Patients initially diagnosed with either MCS− or MCS+ who progress to eMCS following therapeutic intervention. The clinical effective: Patients initially diagnosed with MCS− who progress to MCS+ status following therapeutic intervention. The clinical ineffective: Characterized by either (a) patients initially diagnosed with MCS− who regress to vegetative state (VS) or maintain MCS− status following therapeutic intervention; or (b) patients initially diagnosed with MCS+ who regress to VS, MCS-, or maintain MCS+ status without further progression following therapeutic intervention.

#### Awakening effective rate and awakening time (Using the CRS-R scale as the reference standard)

Awakening effective rate = The number of awakening cases in each group ÷ The total number of persons in each group × 100%. The number of awakening cases: The state of consciousness after treatment is improved compared with that before treatment. For example, patients initially diagnosed with MCS− who progress to either MCS+ or eMCS following therapeutic intervention, as well as patients initially diagnosed with MCS+ who progress to eMCS following therapeutic intervention. Awakening time is the point at which the patient exhibits improvement in the type of consciousness described above.

### Safety assessments

In this study, all adverse events, including acupuncture-related adverse reactions and serious adverse events, will be recorded and evaluated in detail in the Case Report Form, and corresponding laboratory test results will be used to assess the safety of acupuncture treatment.

Some patients in the study may have experienced local symptoms such as skin irritation, commonly including local erythema, desquamation, dryness, itching, hyperpigmentation at the site of medication, hirsutism, capillary dilatation, and skin atrophy, which will be assessed and recorded once every 2 weeks during the observation. The adverse reactions to acupuncture include fainting needles, broken needles, bending needles, subcutaneous hematoma, etc., which will be recorded once after each treatment. Laboratory safety tests will be recorded once before treatment and once at the end of the disease course. Laboratory abnormalities will be documented and will require thorough assessment to determine potential causal relationships with acupuncture intervention. Serious adverse events such as pneumothorax and needle site infection should be reported to the person in charge of the research center, the person in charge of the research group, and the contact person in time. In conducting these recordings and assessments, the researchers will follow strict data recording and reporting procedures to ensure the accuracy and completeness of all the information. In addition, all documented adverse events will be analyzed in detail to determine their causal relationship with acupuncture treatment, and appropriate measures will be taken to prevent and manage these adverse events.

### Sample size calculation

The GCS score was used as the primary outcome measure for the sample size calculation, following the method outlined by Lai Shilong in the Methodology of Clinical Research of Integrated Chinese and Western Medicine. Based on a study by Li Yuyuan et al. (25), the GCS score for the acupuncture combined with rTMS group was 10.30 ± 3.47 after 4 weeks of treatment. The GCS score for the acupuncture-only group was 8.33 ± 2.97, whereas that for the rTMS group was 8.16 ± 2.98. Taking *α* = 0.05, two-sided test, and *β* = 0.2, the ratio of the three groups of sample sizes was 1:1:1. After the above values are substituted with PASS 15 software, the sample size required for each group is calculated to be 32 cases. Considering a shedding rate of 20%, 39 patients are included in each group, and 117 patients are ultimately included in this study.

### Data collection and management

Personnel involved in data recording and evaluation will undergo comprehensive training in clinical research methodology and data management to ensure data quality, including accuracy, integrity, consistency, and timeliness throughout the collection process. The standardized process will be followed to collect data, check the input data, and ensure the quality of the data. If problems in data verification are identified, they will communicate with the researchers in time and record the question and answer process. These evaluation criteria and processes will provide reliable research support.

### Statistical analysis

First, ANOVA analysis assumes that all continuous variables satisfy the normality and homogeneity of variance. If the test finds a violation of the assumption, a non-parametric alternative method will be used. For repeated measurement data (such as fMRI), the spherical hypothesis is satisfied by default. And if the hypothesis is found to be violated, the Greenhouse–Geisser will be used to correct the degrees of freedom. All analyses follow the intention-to-treat (ITT) principle, and the missing data are carried out by the last observation carried forward (LOCF).

#### Clinical data analysis

SPSS 25.0 software will be used for data analysis. The measurement data will be normally distributed and expressed as mean ± standard deviation ( $\bar{x}$ ± *s*). Paired *t*-test will be used for comparisons between groups before and after treatment. One-way analysis of variance will be used for the comparison of differences between the three groups, and the *q*-test will be used for comparisons between groups. The *χ*^2^-test will be used for pairwise comparisons of enumeration data, and the Mann–Whitney test and Wallis rank sum test will be used for comparisons among the three groups. *p* < 0.05 will be considered statistically significant.

#### MRI data analysis

The MRI data are preprocessed using the SPM8 software platform (SPM8, Wellcome Department of Imaging Neuroscience, London, United Kingdom). Referring to the study of resting-state connectivity of DLPFC in PSD patients (32), we select DLPFC as the seed point, use WFU Pickatlas (33) to define the region of interest (ROI) in CCN, and perform whole brain regression analysis. The resting state connectivity of DLPFC in CCN is compared among the three groups, and the brain changes of each group are evaluated by paired t-test through intra-group analysis (before and after treatment). Covariates such as age and gender are included in the data analysis. The Pearson correlation coefficient is used to analyze the relationship between the improvement of the scale and the change of fMRI image data.

### Patient and public involvement

This study protocol does not incorporate patient or public involvement in the design, implementation, or selection of outcome measures. Assessment of intervention burden from the patient perspective will not be conducted within the scope of this investigation. The results of this study will be disseminated to study participants via our hospital’s website.

### Ethics and dissemination

The Ethics Committee of the Second Chinese Medicine Hospital of Guangdong Province believes that this study is in line with the principles of the “Helsinki Declaration” and meets the requirements of medical ethics. The study was successfully reviewed by the hospital ethics committee (Research Ethics Review Approval Number: K202405-003-01). In addition, this study was registered on the Chinese Clinical Trial Registry (CHICTR) platform. (Clinical Trial Registration Number: ChiCTR2400086163.) In this study, each patient will be provided with sufficient information about the purpose of the study, possible risks, and benefits. Before enrollment, the informed consent documentation will be thoroughly explained to patients’ legal representative by the investigator, after which authorized family members will sign written informed consent in accordance with institutional ethics requirements. After the completion of the trial, the statistical results will be made public and published in peer-reviewed journals.

## Discussion

According to global epidemiological surveys (23, 34), stroke is the second leading cause of disability and death worldwide, and stroke has grown to be the leading cause of death from disease in China with an increasing incidence in younger populations (35). The MCS is a serious poststroke disturbance of consciousness. It refers to the patient’s ability to retain a part of consciousness and attention to the outside world and himself, with a clear but limited, tiny cognitive self and the surrounding environment. The emergence of MCS signifies a positive prognostic development, bringing hope for more patients with disturbance of consciousness to continue treatment. This single-center design, third-party, blinded, randomized controlled trial will test the curative effect of the acupuncture for arousal combined with rTMS and provide a more clinical basis for the awakening treatment of disturbance of consciousness in the future.

According to the theory of traditional Chinese medicine, stroke is located in the brain. Acupuncture for arousal improves the level of consciousness by acupuncture at the head and neck acupoints. The acupoints selected by the acupuncture for arousal are located in the Governor Vessel (GV), the Gallbladder Meridian of Foot Shaoyang (GB), and the Bladder Meridian of Foot Taiyang (BL), which are the key parts of the meridian circulation in the brain. This is in line with the principle that “where the meridian passes, the main points reach.” The selected acupoints are bilateral GB20 (Fengchi), GB12 (Wangu), BL10 (Tianzhu), GV15 (Yamen), and GV16 (Fengfu). Among them, GB20 can promote the blood circulation of peripheral nerve blood vessels, increase the blood flow rate, relieve vasospasm, and improve the level of ischemia and hypoxia in brain cells (36). Research has shown that acupuncture at GB12 can improve the blood supply of the vertebral–basilar artery and promote the formation of cerebral collateral circulation (37, 38). BL10 can regulate the qi and blood of the head meridians and regulate the function of the cerebral cortex (39). GV15 and GV16 are selected for the Governor Vessel, which dredges the governor vessel and regulates spirit, opening the orifices. After acupuncture, it can promote the activity of the plasma fibrinolytic system, which is beneficial for the dissolution and absorption of blood clots in cerebral hemorrhages and promotes the recovery of comatose patients (40, 41). Furthermore, rTMS is a clinically recognized physical wake-up treatment. Its therapeutic target is in a specific cortical functional area, which can activate or inhibit the activity of cortical–cortical and cortical–subcortical neural networks (17) and regulate the plasticity of the cortex (42), thereby achieving perceptual remodeling. The pathological mechanism of stroke is related to the activity of the *α*-amino-3-hydroxy-5-methyl-4-isoxazole propionate (AMPA) receptor. AMPA receptor is a glutamate receptor that mediates rapid excitatory synaptic transmission in the central nervous system. It is involved in synaptic plasticity and is closely related to neural development, learning and memory (43). However, its excessive activation has a strong excitatory toxicity. rTMS may play a role by inhibiting the over-expression of AMPA receptor (44).

Acupuncture is still based on a summary of clinical experience, and there are various problems in research design. Because coma patients cannot sit autonomously, traditional acupuncture treatment generally involves patients in the supine position. Owing to their position, fewer neck acupoints are selected. This study selects head and neck acupoints and innovatively selects the acupuncture position in which the head of the patient is fixed by a nursing worker or family member to maintain the sitting position. In the previous clinical trial, this study found that acupuncture at neck acupoints can improve the blood supply of the vertebral artery and carotid artery, and the neck acupoints selected by this acupuncture method are close to the medulla oblongata and brainstem reticular structure. Acupuncture may improve the signal of the brainstem reticular ascending activation system, improve the blood supply of the brain, and enhance the compensatory mechanism of the brain. In addition, a sitting position can induce the emergence of proprioception in patients, which can achieve a better curative effect. In addition to the use of the most common behavioral assessment scale, such as the GCS, this study also adds the CRS-R score to accurately determine the level of minimum consciousness and combines objective-specific neurological function laboratory indicators to improve the objectivity and accuracy of the assessment. A follow-up at 4 weeks after the end of treatment will be added to verify that the study had long-term efficacy. NSE and S-100B are specific markers of the central nervous system. Clinical studies have demonstrated elevated NSE levels in both cerebrospinal fluid and peripheral blood shortly after stroke onset (45, 46),while increased S-100B protein concentrations have been consistently observed in cerebrospinal fluid of stroke patients (47, 48). And the degree of cognitive dysfunction in stroke patients is positively correlated with the expression of NSE and S-100B (49). Xu et al. (50) have also confirmed that the expression of NSE and S-100B in the serum of stroke patients is up-regulated after stroke, and the expression is down-regulated after treatment, reflecting that NSE and S-100B can help determine the prognosis of stroke. Therefore, NSE and S-100B will be used as one of the outcome indicators to help verify whether this study has therapeutic significance through the values changes before and after treatment. A reduction in post-treatment NSE and S-100B levels compared to baseline values will indicate therapeutic significance. Brain functional magnetic resonance imaging is an advanced brain function examination tool that can be accurately positioned clinically. BOLD-fMRI reflects neural activity by detecting changes in blood oxygen in the brain, and provides a visual research method for studying changes in brain function (51). However, in the current research on the brain effect of acupuncture based on BOLD-fMRI, Taixi (KI3), Hegu (LI4) and other limb acupoints are the most studied acupoints (52, 53). This study will select head and neck acupoints and use BOLD-fMRI to observe the brain effects during acupuncture objectively and stereoscopically, so as to improve the evaluation of the efficacy of patients with MCS, and further explore the mechanism of functional reorganization of brain regions in MCS patients.

However, this study also has several limitations. First, owing to ethical principles, the basic Western medicine treatment of patients still needs to be carried out at the same time, and its impact on efficacy cannot be ruled out, which may affect the evaluation of overall efficacy. But this study also avoids the use of awakening drugs that affect neurological function and drugs that affect brain metabolism as strictly as possible. For example, citicoline can improve cognition by reducing oxidative stress and increasing antioxidant capacity (54). Trimetazidine improves brain activity by improving brain glucose uptake, anti-oxidation and regulating AMPA metabolism (55). Second, efficacy assessment employs a third-party blind evaluation methodology conducted by professionally trained evaluators. While these evaluators remain unaware of treatment group assignments and are not involved in the intervention delivery, we acknowledge that some element of subjectivity may persist in the assessment process despite these methodological safeguards. In addition, the acupoints selected in this study are in the neck, and electrical stimulation treatment is performed. The general acupuncture needles and electroacupuncture stimulators cannot enter the magnetic resonance machine room, so it is impossible to perform a BOLD-fMRI examination at the same time. Moreover, the data processing of fMRI mainly focuses on the analysis of the dorsolateral prefrontal lobe. In the future, research on other related brain regions can be expanded further to explore the mechanism of functional reorganization of brain regions. Therefore, in future research, we should actively apply for more funds, optimize the experimental design scheme, and improve the imaging examination skills to supplement this research. Finally, since this study is a single-center study conducted only in the Second Chinese Medicine Hospital of Guangdong Province, the results of our study may have limitations: while our enrolled patient population represents the demographic profile and disease spectrum typically encountered in our locale, these characteristics may not fully reflect patient populations in other geographic regions. Additionally, despite our development of detailed procedural guidelines to enhance methodological reproducibility, potential variations in technical execution of TCM interventions cannot be entirely eliminated. However, it cannot be denied that the single-center design has advantages in controlling variables and ensuring treatment consistency. Therefore, even with these limitations, this study provides valuable evidence for the feasibility of acupuncture for arousal combined with rTMS in the treatment of MCS patients, and provides more experience for future research in this field. In future studies, we hope to carry out multi-center randomized controlled trials in a number of hospitals in different regions (including Western hospitals) to verify the broad applicability of our research results.
